# Inequity in out-of-pocket payments for hospitalisation in India: Evidence from the National Sample Surveys, 1995–2014

**DOI:** 10.1016/j.socscimed.2018.01.031

**Published:** 2018-03

**Authors:** Anamika Pandey, Lynda Clarke, Lalit Dandona, George B. Ploubidis

**Affiliations:** a Public Health Foundation of India, National Capital Region, Gurugram, India; b Department of Population Health, London School of Hygiene & Tropical Medicine, London, UK; c Institute for Health Metrics and Evaluation, University of Washington, Seattle, WA, USA; d Centre for Longitudinal Studies, Department of Social Science, UCL - Institute of Education, University College London, UK

**Keywords:** Gender, Horizontal inequity, Hospitalisation, Less developed states, Older population, Out-of-pocket payments, Regressive, Vertical inequity

## Abstract

**Objective:**

We report inequity in out-of-pocket payments (OOPP) for hospitalisation in India between 1995 and 2014 contrasting older population (60 years or more) with a population under 60 years (younger population).

**Methods:**

We used data from nationwide healthcare surveys conducted in India by the National Sample Survey Organisation in 1995–96, 2004 and 2014 with the sample sizes ranging from 333,104 to 629,888. We used generalised linear and fractional response models to study the determinants of OOPP and their burden (share of OOPP in household consumption expenditure) at a constant price. The relationship between predicted OOPP and its burden with monthly per capita consumption expenditure (MPCE) quintiles and selected socioeconomic characteristics were used to examine vertical and horizontal inequities in OOPP.

**Results:**

The older population had higher OOPP for hospitalisation at all time points (range: 1.15–1.48 times) and a greater increase between 1995–96 and 2014 than the younger population (2.43 vs 1.88 times). Between 1995–96 and 2014, the increase in predicted mean OOPP for hospitalisation was higher for the poorest than the richest (3.38 vs 1.85 times) older population. The increase in predicted mean OOPP was higher for the poorest (2.32 vs 1.46 times) and poor (2.87 vs 1.05 times) older population between 1995–96 and 2004 than in the latter decade. In 2014, across all MPCE quintiles, the burden of OOPP was higher for the less developed states, females, private hospitals, and non-communicable disease and injuries, more so for the older than the younger population. In 2014, the predicted absolute OOPP for hospitalisation was positively associated with MPCE quintiles; however, the burden of OOPP was negatively associated with MPCE quintiles indicating a regressive system of healthcare financing.

**Conclusion:**

High OOPP for hospitalisation and greater inequity among older population calls for better risk pooling and prepayment mechanisms in India.

## Highlights

• Older population had higher OOPP for hospitalisation and a greater increase.
• Inequities in OOPP for hospitalisation was higher for the older population in 2014.
• OOPP for hospitalisation was regressive in 2014.
• Gap in the OOPP between private and public hospitals increased by 2014.
• OOPP for NCD hospitalisation increased most for the poorest by 2014.

## 1 Introduction

Achieving equity in the delivery of healthcare, protection from the risk of financial loss and attaining fairness in the distribution of the financing burden are the fundamental goals of healthcare systems. Equitable financing, based on the premise that the risk each household faces due to the costs of the healthcare is distributed according to the ability to pay rather than to the risk of illness is a key dimension of health system's performance (World Health Organization, 2000). Financial protection is also the key element of Universal Health Coverage which aims at ensuring health services for people without the risk of financial catastrophe (World Health Organization, 2010). The increasing dependence on private care with an absence of adequate medical insurance and increasing cost of medical care are some of the principal causes of direct debt and poverty in India (Balarajan et al., 2011). Catastrophic healthcare expenditures are a major cause of household debt for families on low and middle incomes; indeed, the cost of healthcare is a leading cause of poverty in India (David et al., 2001, Van Doorslaer et al, 2006, Garg and Karan, 2009, Shahrawat and Rao, 2012). Annually, about 7 percent of the population in India is pushed below the poverty line due to the out-of-pocket payments (OOPP) for healthcare alone (Kumar et al., 2015).

India's health system ranks as one of the most heavily dependent on out-of-pocket (OOP) expenditure in the world (Reddy et al., 2011). High proportions of OOPP for healthcare can keep a country from attaining equitable financing because OOPP for healthcare tends to be regressive and often impede access to health services (World Health Organization, 2000). Evidence suggests that the healthcare cost in India has become more impoverishing than ever before and almost all hospitalisations, even in public facilities lead to catastrophic health expenditures (Government of India and National health policy draft., 2014). Over the past decade in India, the expenditure on outpatient care increased more than 100 percent while the expenditure on inpatient care increased by almost 300 percent (Jayakrishnan et al., 2016). Moreover, the healthcare expenditure for the older population is found to be considerably higher than other age groups and the concerns over high OOP expenditures are greatest for this group (Kim et al, 2005, Mohanty et al, 2013, Mohanty et al, 2016, Kumara and Samaratunge, 2016, Baird, 2016). It is of immense importance from a policy perspective to obtain evidence on the inequities in OOPP for hospitalisation of the older population in India, given their increasing proportion in the total population, higher disease burden, increasingly higher cost of healthcare and persistently low public investment in healthcare.

This study is the first of its kind to compare the horizontal and vertical inequities in OOPP for hospitalisation of the older population (60 years or more) with the population under 60 years (younger population) in India in 1995–96, 2004 and 2014 using national wide healthcare surveys.

## 2 Methods

### 2.1 Data

We used individual-level data from three rounds of the National Sample Survey Organisation (NSSO): survey on healthcare of 1995–96 (52^nd^ round); survey on morbidity and healthcare of 2004 (60^th^ round); and survey on social consumption: health of 2014 (71^st^ round) conducted under the stewardship of the Ministry of Statistics and Programme Implementation, Government of India. Details of the sampling design, survey instruments, and initial findings can be found in the national reports (Ministry of Statistics and Programme Implementation, 1998, Ministry of Statistics and Programme Implementation, 2004, Ministry of Statistics and Programme Implementation, 2014). All the surveys collected detailed information on the expenditure incurred on each episode of hospitalisation within a 365-days reference period. NSS 1995–96 was a full year survey done in four sub-rounds (July 1995–June 1996), whereas, NSS 2004 and NSS 2014 were half year surveys done in two sub-rounds between January and June. We used full year NSS 1995–96 survey for this analysis. For robustness check, we compared data from the two sub-rounds of NSS 1995–96 conducted between January and June 1996 which corresponds to the survey period of NSS 2004 and NSS 2014 with the full year NSS 1995–96 survey. The predicted mean annual out-of-pocket payments from the half year NSS 1995–96 survey were generally similar to the estimates obtained using all the four sub-rounds; the 95% confidence intervals (95% CI) for most estimates were overlapping (Appendix Table 1). We limit our analysis to the older population who were hospitalised at least once during the 365-days reference period and were alive at the time of survey with sample sizes 3,209 in NSS 1995–96; 4,974 in NSS 2004 and 7,065 in NSS 2014. For comparison purposes, we present results of the hospitalised population under 60 years with sample sizes: 19,597 in NSS 1995–96; 24,062 in NSS 2004 and 28,606 in NSS 2014.

### 2.2 Dependent variables

Our dependent variable was the OOPP made on all episodes of hospitalisation by an individual and the ratio of individual OOPP on hospitalisation in total household consumption expenditure, henceforth called the burden of OOPP. We exclude from individuals' OOP expenses any payments that were later reimbursed by employers/other agencies. The expenditure on hospitalisation includes doctor's/surgeon's fee, bed charges, cost of medicines, charges for diagnostics tests, charges for ambulance and other services, cost of oxygen and blood supply, attendant charges, cost of personal medical appliances, physiotherapy, food and other materials, transportation other than ambulance and lodging charges of the escorts. The expenditure reported in Indian rupees (INR) were converted to 2014 prices using the gross domestic product (GDP) deflator and then to United States dollars (US$; exchange rate: US$ 1 = 63.33 INR) (International Monetary Fund, 2016a, International Monetary Fund, 2016b). As the consumer price index could be an alternate method of deflating, we also checked how the estimates for the change in OOPP for hospitalisation from NSS 1995–96 to NSS 2014 would compare with those using the GDP deflator (International Monetary Fund, 2016a). The use of GDP deflator produced a somewhat higher increase in the mean annual OOPP for hospitalisation than the consumer price index, but the trends were quite similar (Appendix Table 2).

### 2.3 Covariates

Information on household consumption expenditure was available in these surveys only in aggregate in the 30-days reference period. We converted the consumption expenditure to correspond to the same recall period to make them comparable with OOPP for hospitalisation. We used household consumption expenditure adjusted for household size and economies of scale as a measure of economic status (Deaton, 1997). Based on the Andersen's model of healthcare utilisation we identified, age, sex, marital status and social group as predisposing factors, monthly per capita consumption expenditure (MPCE) quintiles, education, rural/urban, and less/more developed states as enabling factors, and whether hospitalised more than once, whether hospitalised at least once in private hospital and whether hospitalised at least once for non-communicable diseases and injuries (NCDs) as the need factors (Andersen, 2008).

### 2.4 Statistical analysis

To model individual OOPP for hospitalisation we used a generalised linear model with gamma distribution and log link function to take into account the positive skewness in the expenditure data (Manning et al., 2005). The output was presented as exponentiated coefficients with 95% CI for NSS 1995–96, NSS 2004 and NSS 2014, separately. In order to analyse the burden of OOPP, a fractional response generalised linear model was used (Papke and Wooldridge, 1996, Papke and Wooldridge, 2008, Gallani et al., 2015). We used a logit link function which is the canonical link function for generalised linear models for the binomial family. This model can predict determinants of proportions and requires a dependent variable ranging from ‘0’ to ‘1’. The share of OOPP in household's consumption expenditure is a proportion. However, it could occur that total OOPP exceeded the consumption expenditure in the preceding 365-days. In these cases, when the dependent variable was greater than ‘1’, the values were replaced by ‘1’ for the regression analysis. The results were reported as average marginal effects with robust standard errors for NSS 1995–96, NSS 2004 and NSS 2014, separately. We used P-values for the Wald test to assess the difference in magnitude of coefficients between NSS 1995–96 and NSS 2014.

To assess vertical inequities (similar out-of-pocket payments by households with unequal ability to pay), we examined how predicted OOPP for hospitalisation, both absolute and as a share of household consumption expenditure varied across MPCE quintiles. Mean predicted OOP expenditure and shares were calculated across MPCE quintiles, setting all other covariates at their sample means. To assess horizontal inequities (dissimilar out-of-pocket payments by households with equal ability to pay), we compared whether predicted OOPP, both absolute and as a share of household consumption expenditure, varied among individuals across two groups distinguished by a non-income-related characteristic, but were otherwise similar in terms of MPCE quintiles and other non-income-related characteristics. The non-income-related characteristics that were varied to assess horizontal inequities across the two groups were gender (male vs female), place of residence (rural vs urban), state (less developed vs more developed states), whether hospitalised in private hospital (yes vs no) and whether hospitalised for NCDs (yes vs no) controlling for the MPCE quintiles and all other non-income-related characteristics that might affect household consumption expenditure. Mean predicted payment shares (burden of OOPP) across adult equivalent MPCE quintiles were obtained by setting the relevant non-income-related characteristics to zero or one (instead of the sample average) and all other covariates at their sample mean. We report 95% CI for the mean predicted OOPP and mean predicted payment shares. The regression-based method for assessing inequities in healthcare cost used here is in line with the previous studies (Roy and Howard, 2007, Chaudhuri and Roy, 2008). We carried all analyses at individual level and applied survey sampling weights.

## 3 Results

### 3.1 Sample characteristics

The proportion of older population hospitalised in 365-days reference period showed a steady increase; from 3.8% in 1995–96 to 8.0% in 2014. The older population in the higher MPCE quintiles reported higher hospitalisation, particularly for NCDs and a greater use of private hospitals in all the three surveys. The increase in mean annual OOPP for hospitalisation was higher than the increase in mean annual household consumption expenditure per capita, more so for the poorest older population (4.60 vs 1.25 times). Higher proportion of the older population in the lower quintiles were illiterate and lived in rural areas (Table 1).

**Table 1:** Selected socio-economic characteristics by monthly per capita consumption expenditure quintiles for hospitalised older population in India, NSS 1995–96, NSS 2004 and NSS 2014.

	Poorest	Poor	Middle	Rich	Richest	All
**NSS 1995**–**96**						
Female (%)	38.0	43.3	35.3	44.3	38.9	40.2
Illiterate (%)	76.1	65.1	65.8	55.1	42.2	54.9
Rural (%)	79.6	82.1	76.1	73.1	53.0	67.7
Less developed states^a^ (%)	32.4	28.0	27.2	24.0	20.5	24.4
Hospitalised for NCDs^b^ (%)	58.2	48.7	54.5	62.4	64.7	60.1
Used private hospital (%)	39.7	46.3	52.8	50.6	74.8	58.5
Mean annual OOPP (US$) per hospitalised person (SD)	40 (76)	40 (46)	100 (112)	118 (131)	328 (488)	179 (334)
Mean annual household consumption expenditure (US$) per capita (SD)	180 (31)	248 (15)	307 (20)	388 (31)	712 (348)	463 (294)
Hospitalised (%)	1.6	2.1	3.0	5.0	7.7	3.8
**NSS 2004**						
Female (%)	42.3	41.0	45.5	44.1	46.7	44.5
Illiterate (%)	77.3	63.3	61.4	50.6	28.7	51.0
Rural (%)	91.1	85.8	80.0	65.8	38.8	66.1
Less developed states^a^ (%)	30.6	33.6	28.1	25.5	19.5	25.9
Hospitalised for NCDs^b^ (%)	71.9	68.2	68.6	67.1	76.1	71.1
Used private hospital (%)	39.9	52.8	60.4	65.1	78.1	63.1
Mean annual OOPP (US$) per hospitalised person (SD)	110 (210)	150 (257)	177 (323)	248 (464)	477 (1,157)	276 (730)
Mean annual household consumption expenditure (US$) per capita (SD)	163 (31)	237 (17)	302 (20)	397 (36)	816 (537)	459 (398)
Hospitalised (%)	3.8	4.6	5.7	7.2	9.8	6.1
**NSS 2014**						
Female (%)	54.0	50.3	49.9	45.2	50.5	49.8
Illiterate (%)	71.7	67.3	63.5	45.6	28.5	49.8
Rural (%)	83.4	78.1	75.3	62.5	38.7	61.9
Less developed states^a^ (%)	53.6	39.8	31.2	27.8	20.4	31.2
Hospitalised for NCDs^b^ (%)	80.7	78.1	75.5	80.2	86.1	81.1
Used private hospital (%)	42.2	52.5	63.6	66.5	81.0	65.5
Mean annual OOP payment (US$) per hospitalised person (SD)	186 (354)	180 (430)	254 (437)	371 (714)	779 (1,454)	435 (979)
Mean annual household consumption expenditure (US$) per capita (SD)	224 (47)	331 (26)	425 (30)	566 (54)	1,199 (734)	674 (578)
Hospitalised (%)	4.9	6.2	7.9	8.5	12.3	8.0

a Includes eight empowered action group states (Bihar, Jharkhand, Madhya Pradesh, Chhattisgarh, Uttar Pradesh, Uttaranchal, Odisha and Rajasthan), 8 north-eastern states (Assam, Arunachal Pradesh, Manipur, Mizoram, Meghalaya, Nagaland, Sikkim, Tripura), Himachal Pradesh and Jammu and Kashmir.

b Based on Global Burden of Disease (2013) classification; NSS: National Sample Survey; NCDs: Non-communicable diseases and injuries; OOPP: Out-of-pocket payments; SD: Standard deviation.

Compared with the younger population, the older people had higher hospitalisation for NCDs (range, 1.46–1.78 times) and consequently higher OOPP (range, 1.15–1.48 times) at all time points. Also, the increase in OOPP for hospitalisation between 1995–96 and 2014 was higher for the older population than the population under 60 years (2.43 vs 1.88 times) (Appendix Table 3).

### 3.2 Determinants of OOPP for hospitalisation

Economic status measured in terms of MPCE quintile was a significant predictor of OOPP after controlling for all other covariates (Table 2). Compared to the richer quintiles, the poorest quintile of the older population had 0.171 times (95% CI 0.125, 0.235) and 0.388 times (95% CI 0.305, 0.493) lower OOPP in 1995–96 and 2014, respectively. The older population residing in less developed states had 42.9% (95% CI 1.205, 1.694) higher mean OOPP than their counterparts in more developed states in 1995–96 which increased to 67.4% (95% CI 1.402, 2.000) in 2014 (P-value = 0.209). Longer duration of hospitalisation significantly increased the mean cost by 5.7% in 2014. Hospitalisation for NCDs was significantly associated with higher mean OOPP and this increased from 21.8% in 1995–96 to 72.0% in 2014 (P-value<0.001). Those hospitalised in private hospitals incurred 2.147 (95% CI 1.828, 2.522) times higher OOPP in 1995–96 which increased significantly to 3.602 (95% CI 3.004, 4.320) times in 2014. Residing in rural areas, being female, illiterate and belonging to the SC/ST social group were associated significantly with lower OOPP in 2014.

**Table 2:** Determinants of out-of-pocket payments for hospitalisation among older population in India, NSS 1995–96, NSS 2004 and NSS 2014.

Background variables	GLM exp(β) (95% CI)			β_NSS 2014_ - β_NSS 1995–96_	p-Value for Wald test (β_NSS 2014_ - β_NSS 1995–96_)
	NSS 1995–96	NSS 2004	NSS 2014		
**MPCE quintile (ref.** = **Richest)**					
Poorest	0.171 (0.125,0.235)	0.371 (0.304,0.452)	0.388 (0.305,0.493)	0.819	<0.001
Poor	0.193 (0.154,0.241)	0.467 (0.390,0.560)	0.373 (0.299,0.466)	0.661	<0.001
Middle	0.362 (0.287,0.457)	0.472 (0.403,0.553)	0.543 (0.448,0.657)	0.406	0.008
Rich	0.478 (0.401,0.570)	0.605 (0.521,0.702)	0.584 (0.488,0.700)	0.201	0.119
**Age (years)**	1.000 (0.989,1.012)	0.993 (0.986,0.999)	1.002 (0.990,1.014)	0.001	0.865
**Gender (ref.** = **Male)**					
Female	0.876 (0.742,1.035)	0.995 (0.865,1.144)	0.845 (0.744,0.959)	−0.037	0.731
**Marital status (ref.** = **Married)**					
Single	0.864 (0.745,1.002)	0.813 (0.709,0.933)	0.822 (0.722,0.937)	−0.049	0.628
**Social group (ref.** = **Non-SC/STs)**					
SC/STs	1.040 (0.865,1.250)	0.816 (0.714,0.933)	0.777 (0.694,0.871)	−0.291	0.009
**Education (ref.** = **Literate)**					
Illiterate	0.862 (0.726,1.022)	0.759 (0.670,0.859)	0.802 (0.723,0.889)	−0.072	0.480
**Place of residence (ref.** = **Urban)**					
Rural	0.953 (0.827,1.098)	0.910 (0.802,1.032)	0.819 (0.716,0.937)	−0.152	0.128
**State group (ref.** = **More developed states)**					
Less developed states	1.429 (1.205,1.696)	1.505 (1.344,1.686)	1.674 (1.402,2.000)	0.158	0.209
**Whether hospitalised more than once (ref. = No)**					
Yes	1.183 (0.950,1.473)	1.369 (1.083,1.730)	1.041 (0.904,1.199)	−0.128	0.337
**Duration of stay in hospital (days)**	1.013 (1.005,1.022)	1.044 (1.036,1.053)	1.057 (1.047,1.067)	0.042	<0.001
**Whether hospitalised for NCDs (ref.** = **No)**					
Yes	1.218 (1.062,1.397)	1.335 (1.196,1.490)	1.720 (1.524,1.942)	0.345	<0.001
**Whether hospitalised in private hospital (ref.** = **No)**					
Yes	2.147 (1.828,2.522)	2.750 (2.402,3.149)	3.602 (3.004,4.320)	0.517	<0.001
Constant	117 (51,268)	151 (89,254)	79 (42,148)	−0.395	0.458
N	3,139	4,913	7,062		

OOPP: Out-of-pocket payments; NSS: National Sample Survey; CI: Confidence interval; MPCE: Monthly per capita consumption expenditure; NCDs: Non-communicable diseases and injuries; SC/STs: Scheduled castes and scheduled tribes.

### 3.3 Determinants of financial burden of OOPP for hospitalisation

Compared to the richest, the poorest older population had 12.2 percentage points higher share of OOPP in their total household consumption expenditure in 2014 (P-value<0.001) (Table 3). Those hospitalised for NCDs had a significantly higher burden of OOPP ranging from 2.8 to 7.4 percentage points between 1995 and 2014. The burden of OOPP was higher in the private than the public hospitals ranging from 7.0 percentage points in 1995–96 to 18.0 percentage points in 2014. The increase in the duration of stay by one day was associated with 0.60 percentage points increase in the burden of OOPP in 2014. Being female and belonging to the SC/ST caste group was significantly associated with a lower burden (by 3.2 percentage points) of OOPP in 2014. Compared to the more developed, those residing in the less developed states had 6.9 percentage points (95% CI 0.033, 0.105) higher burden of OOPP in 2014. The rural residents had 1.80 percentage points significantly higher burden of OOPP in 1995–96 than the urban residents. Literacy was significantly associated with the burden of OOPP, but only in 2004, where the illiterate older population had 3.1 percentage points lower financial burden.

**Table 3:** Determinants of out-of-pocket payments for hospitalisation as a share of household consumption expenditure for the older population in India, NSS 1995–96, NSS 2004 and NSS 2014.

Background variables	Average partial effects (95% CI)			β_NSS 2014_ - β_NSS 1995–96_	p-Value for Wald test (β_NSS 2014_ - β_NSS 1995–96_)
	NSS 1995–96	NSS 2004	NSS 2014		
**MPCE quintile (ref.** = **Richest)**					
Poorest	−0.052 (−0.083,−0.020)	0.122 (0.086,0.159)	0.122 (0.071,0.173)	1.322	<0.001
Poor	−0.073 (−0.092,−0.055)	0.075 (0.046,0.103)	0.013 (−0.033,0.058)	0.973	<0.001
Middle	−0.014 (−0.068,0.040)	0.038 (0.015,0.061)	0.029 (−0.009,0.066)	0.337	0.261
Rich	−0.034 (−0.054,−0.014)	0.019 (−0.001,0.039)	0.007 (−0.027,0.041)	0.385	0.018
**Age (years)**	−0.001 (−0.002,0.001)	−0.001 (−0.002,0.000)	−0.001 (−0.003,0.000)	−0.002	0.821
**Gender (ref.** = **Male)**					
Female	−0.031 (−0.055,−0.007)	−0.011 (−0.030,0.009)	−0.032 (−0.053,−0.012)	0.107	0.459
**Marital status (ref.** = **Married)**					
Single	−0.005 (−0.024,0.014)	−0.011 (−0.031,0.008)	0.004 (−0.022,0.031)	0.084	0.543
**Social group (ref.** = **Non-SC/STs)**					
SC/STs	0.003 (−0.024,0.030)	−0.033 (−0.053,−0.013)	−0.032 (−0.051,−0.012)	−0.258	0.102
**Education (ref.** = **Literate)**					
Illiterate	0.007 (−0.022,0.035)	−0.031 (−0.050,−0.012)	−0.016 (−0.037,0.005)	−0.178	0.285
**Place of residence (ref.** = **Urban)**					
Rural	0.018 (0.003,0.033)	0.006 (−0.013,0.024)	−0.013 (−0.038,0.012)	−0.283	0.018
**State group (ref.** = **More developed states)**					
Less developed states	0.007 (−0.015,0.028)	0.033 (0.016,0.050)	0.069 (0.033,0.105)	0.389	0.014
**Whether hospitalised more than once (ref.** = **No)**					
Yes	0.032 (0.000,0.064)	0.027 (0.000,0.055)	0.033 (0.004,0.061)	−0.088	0.618
**Duration of stay in hospital (days)**	0.000 (0.000,0.001)	0.005 (0.004,0.006)	0.006 (0.005,0.007)	0.040	<0.001
**Whether hospitalised for NCDs (ref.** = **No)**					
Yes	0.028 (0.007,0.049)	0.038 (0.021,0.055)	0.074 (0.055,0.094)	0.267	0.048
**Whether hospitalised in private hospital (ref.** = **No)**					
Yes	0.070 (0.050,0.089)	0.141 (0.126,0.155)	0.180 (0.157,0.202)	0.656	<0.001
N	3,139	4,913	7,062		

OOPP: Out-of-pocket payments; NSS: National Sample Survey; CI: Confidence interval; MPCE: Monthly per capita consumption expenditure; SC/STs: Schedules castes and scheduled tribes; NCDs: Non-communicable diseases and injuries.

### 3.4 Vertical inequities in OOPP for hospitalisation

**Fig. 1:**
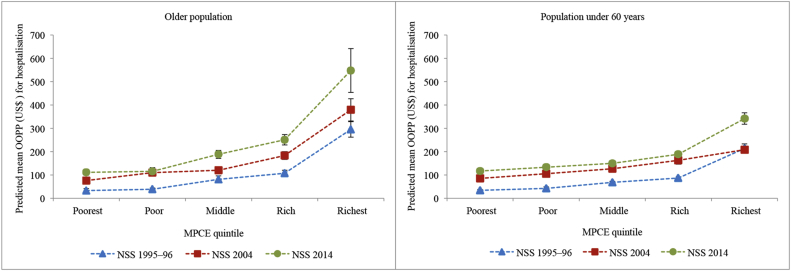
Predicted mean out-of-pocket payments for hospitalisation across monthly per capita consumption expenditure quintiles by age groups in India, NSS 1995–96, NSS 2004 and NSS 2014. OOPP: Out-of-pocket payments; US$: United States dollars; NSS: National Sample Survey; MPCE: Monthly per capita consumption expenditure.

**Fig. 2:**
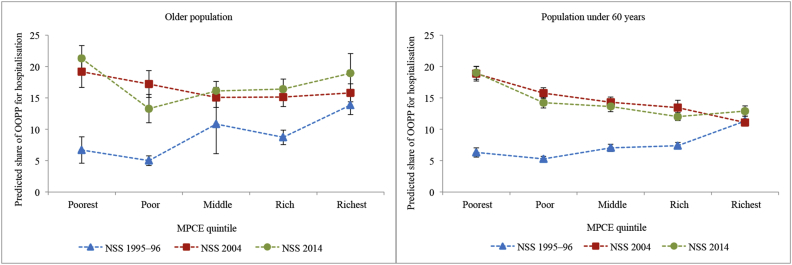
Predicted shares of out-of-pocket payments for hospitalisation in household consumption expenditure across monthly per capita consumption expenditure quintiles by age groups in India, NSS 1995–96, NSS 2004 and NSS 2014. OOPP: Out-of-pocket payments; NSS: National Sample Survey; MPCE: Monthly per capita consumption expenditure.

Fig. 1 shows the trends in predicted mean OOPP for hospitalisation across MPCE quintiles for the older population and the population under 60 years in India. The OOPP increased with the rising household consumption expenditure for all the three surveys. The OOPP of the poorest older population increased 3.38 times in the two decades, while that of the richest increased 1.85 times. The increase in OOPP was higher for the poorest older population between 1995–96 and 2004, while it was higher for the richest in the latter decade. Since payments are expressed in absolute terms, it does not truly assess the “progressivity” (or vertical equity) of the financial system. Fig. 2 captures the latter aspect as it shows the proportion of consumption spent on hospitalisation across the MPCE quintiles over two decades. In 1995–96, the older population in the lower MPCE quintiles paid a lower share, indicating a progressive system, and in 2004 and 2014, the richer quintiles were paying a lower share indicating a regressive system of healthcare financing.

The trends in OOPP and its burden were similar for the two age groups; however, the levels were different. The OOPP of the older population in poor quintiles were similar to those under 60 years, but in the rich quintiles, the older population had higher OOPP than the younger population at all time points (range, 1.12–1.82 times) (Fig. 2).

### 3.5 Horizontal inequities in OOPP for hospitalisation

Table 4 shows that the OOPP for hospitalisation in private hospitals by the older population was considerably higher than that in the public hospitals across quintiles in all the years (range, 46.6–74.9%) with the gap being highest in 2014. The cost of hospitalisation for NCDs was higher than CDs/other diseases across MPCE quintiles in 2004 and 2014 (range, 31.5–58.0%); this difference was higher for the poorest than the richest older population. The OOPP for hospitalisation of the older population was higher in the less developed than the more developed states across all quintiles in 2014; the gap was higher for the poorest than the richest (39.6 vs 15.5%). The rural and urban older population made similar OOPP for hospitalisation across MPCE quintiles at all time points. A substantial difference in OOPP was observed by gender in 2014 with male older population incurring higher OOPP for hospitalisation.

**Table 4:** Predicted mean annual out-of-pocket payments for hospitalisation of the older population and the population under 60 years in India, NSS 1995–96, NSS 2004 and NSS 2014.

Predicted mean annual out-of-pockets (US$) (95% CI)										
MPCE quintile	Male	Female	Urban	Rural	More developed states	Less developed states	Public	Private	CDs/other diseases	NCDs
Older population										

**NSS 1995–96**										
Poorest	36 (26–45)	29 (21–37)	28 (20–36)	34 (25–43)	31 (23–38)	38 (26–50)	25 (18–31)	49 (35–64)	28 (20–36)	37 (28–46)
Poor	45 (38–52)	31 (26–36)	35 (29–41)	39 (33–45)	37 (31–43)	41 (34–49)	29 (24–33)	54 (45–63)	35 (29–40)	42 (35–49)
Middle	88 (71–105)	70 (56–84)	76 (61–92)	83 (67–98)	79 (64–95)	85 (68–103)	54 (44–65)	116 (92–140)	64 (52–76)	98 (78–119)
Rich	120 (104–137)	92 (80–105)	109 (92–125)	107 (93–120)	102 (88–115)	127 (107–146)	75 (63–87)	149 (129–168)	96 (82–110)	114 (99–130)
Richest	343 (298–387)	233 (200–265)	275 (245–305)	315 (266–364)	288 (256–320)	327 (265–390)	178 (145–211)	350 (312–388)	232 (192–272)	336 (299–372)
**NSS 2004**										
Poorest	77 (64–89)	75 (63–88)	69 (55–83)	77 (65–89)	72 (60–84)	87 (73–101)	53 (44–62)	130 (108–152)	54 (45–63)	87 (73–101)
Poor	127 (109–145)	89 (76–102)	119 (98–139)	108 (94–123)	103 (89–117)	124 (107–141)	63 (54–73)	178 (154–203)	77 (66–89)	130 (113–147)
Middle	136 (120–151)	103 (91–116)	125 (107–143)	119 (106–132)	110 (97–122)	152 (134–170)	67 (58–76)	176 (156–196)	87 (76–98)	139 (124–155)
Rich	208 (186–229)	156 (139–172)	183 (160–206)	183 (164–201)	166 (149–182)	245 (217–273)	99 (87–111)	254 (228–280)	142 (125–158)	207 (186–228)
Richest	422 (372–472)	335 (282–389)	385 (329–441)	370 (321–419)	368 (320–416)	429 (366–493)	189 (157–222)	461 (402–519)	283 (243–323)	416 (361–471)
**NSS 2014**										
Poorest	131 (114–149)	96 (85–108)	113 (95–131)	111 (99–123)	85 (74–97)	140 (121–159)	62 (54–71)	247 (213–281)	55 (48–63)	131 (117–146)
Poor	138 (117–159)	98 (83–112)	130 (106–153)	112 (97–127)	103 (87–118)	139 (114–164)	60 (49–70)	211 (177–245)	67 (57–77)	135 (116–154)
Middle	234 (206–262)	151 (135–167)	217 (186–247)	180 (163–197)	167 (150–184)	245 (206–284)	90 (75–105)	288 (258–318)	110 (97–123)	224 (202–246)
Rich	341 (301–381)	172 (156–189)	276 (242–310)	236 (213–259)	218 (198–237)	360 (297–424)	104 (87–122)	389 (352–426)	126 (111–140)	297 (268–325)
Richest	677 (529–825)	445 (383–507)	583 (463–703)	496 (422–570)	529 (451–607)	627 (439–814)	186 (127–244)	706 (603–810)	272 (226–318)	613 (505–722)
Population under 60 years										
**NSS 1995–96**										
Poorest	36 (31–40)	33 (29–37)	24 (20–27)	37 (33–41)	29 (26–32)	46 (40–51)	26 (23–29)	56 (49–63)	31 (27–34)	46 (40–52)
Poor	48 (41–55)	39 (34–44)	34 (29–39)	45 (39–51)	40 (35–46)	49 (43–56)	33 (28–38)	68 (59–77)	37 (32–42)	63 (55–72)
Middle	73 (67–80)	62 (57–67)	52 (46–57)	73 (68–79)	65 (60–70)	75 (69–82)	50 (46–53)	91 (83–99)	58 (53–62)	97 (87–107)
Rich	92 (85–98)	82 (76–88)	71 (64–77)	95 (88–101)	82 (76–88)	98 (91–106)	64 (59–69)	111 (103–118)	72 (67–77)	129 (118–141)
Richest	229 (202–256)	199 (184–214)	184 (171–197)	250 (218–281)	202 (182–222)	254 (232–276)	148 (135–161)	256 (230–282)	165 (153–177)	316 (273–358)
**NSS 2004**										
Poorest	95 (88–102)	76 (71–82)	77 (69–85)	86 (81–92)	71 (66–77)	106 (99–113)	57 (53–60)	147 (136–157)	65 (60–69)	127 (118–136)
Poor	115 (108–122)	97 (91–102)	102 (92–112)	106 (101–112)	93 (87–98)	132 (124–140)	64 (60–68)	164 (155–174)	76 (71–80)	162 (152–173)
Middle	128 (120–136)	124 (117–132)	115 (105–126)	129 (122–137)	112 (105–119)	158 (149–167)	77 (72–82)	184 (173–195)	93 (87–99)	175 (163–186)
Rich	173 (153–194)	152 (138–166)	145 (125–165)	173 (157–189)	149 (132–166)	197 (179–214)	98 (87–108)	218 (195–241)	118 (104–131)	226 (204–249)
Richest	215 (203–228)	201 (189–214)	194 (183–206)	230 (213–247)	195 (185–205)	261 (244–278)	114 (107–122)	261 (247–275)	147 (139–156)	292 (274–310)
**NSS 2014**										
Poorest	134 (125–143)	104 (97–110)	122 (112–132)	116 (109–123)	105 (97–113)	124 (117–132)	66 (61–70)	258 (240–276)	78 (73–83)	166 (155–177)
Poor	147 (134–160)	121 (109–133)	138 (124–152)	132 (120–144)	128 (118–139)	138 (123–152)	68 (60–75)	254 (232–276)	92 (83–102)	186 (170–202)
Middle	164 (154–174)	137 (129–144)	146 (136–156)	151 (143–159)	143 (135–151)	162 (152–171)	71 (66–76)	251 (237–265)	100 (93–106)	203 (191–214)
Rich	210 (197–224)	170 (159–181)	180 (166–194)	194 (183–205)	181 (170–193)	205 (191–219)	83 (76–90)	288 (272–305)	127 (117–136)	263 (247–278)
Richest	393 (363–423)	301 (277–326)	337 (313–361)	349 (318–381)	329 (304–354)	393 (362–424)	159 (142–177)	416 (388–445)	223 (205–241)	459 (424–494)

MPCE: Monthly per capita consumption expenditure; CI: Confidence interval; CDs: Communicable diseases and nutritional disorders; Other diseases: Includes other diagnosed and undiagnosed ailments; NCDs: Non-communicable diseases and injuries.

Table 5 reveals the horizontal inequities in OOPP across various subgroups in the three time points. The largest difference in the payment shares of the older population was observed across provider type in 1995–96, 2004 and 2014 (range, 3.8–27.5 percentage points); the gap was highest in 2014, more so for the poorest older population. The older population hospitalised for NCDs incurred a higher share of consumption expenditure across MPCE quintiles in 2004 and 2014 (range, 4.3–12.9 percentage points); this difference was higher for the poorest than the richest older population. In 2014 the OOPP share was higher in the less developed than the more developed states only in the poorest (6.9 percentage points) and rich (6.5 percentage points) MPCE quintiles. The predicted payment share was higher for the male than the female older population in the most recent survey (range, 2.1–7.8 percentage points).

**Table 5:** Predicted shares of out-of-pocket payments for hospitalisation in household consumption expenditure for the older population and the population under 60 years in India, NSS 1995–96, NSS 2004 and NSS 2014.

Predicted shares of out-of-pocket payments (95% CI)										
MPCE quintile	Male	Female	Urban	Rural	More developed states	Less developed states	Public	Private	CDs/other diseases	NCDs
Older population										

**NSS 1995–96**										
Poorest	7.7 (5.4–10.0)	5.4 (3.4–7.4)	5.1 (3.4–6.8)	7.2 (4.9–9.5)	6.6 (4.6–8.5)	7.0 (4.3–9.7)	4.9 (3.3–6.5)	10.3 (6.9–13.6)	5.6 (3.5–7.7)	7.6 (5.4–9.9)
Poor	5.9 (5.0–6.8)	4.0 (3.1–4.9)	3.8 (3.0–4.6)	5.3 (4.5–6.1)	5.0 (4.3–5.8)	4.9 (3.9–5.9)	3.6 (2.8–4.3)	7.4 (6.2–8.6)	4.3 (3.3–5.2)	5.9 (4.9–6.9)
Middle	12.1 (6.5–17.7)	8.8 (5.4–12.3)	9.0 (5.4–12.5)	11.5 (6.3–16.7)	11.2 (6.0–16.4)	9.9 (6.1–13.6)	7.3 (4.5–10.1)	15.2 (7.9–22.6)	8.5 (5.3–11.7)	13.2 (6.7–19.7)
Rich	10.1 (8.7–11.4)	7.2 (5.9–8.5)	7.7 (6.4–9.0)	9.1 (7.9–10.4)	8.7 (7.5–9.9)	8.9 (7.1–10.6)	6.0 (4.8–7.1)	12.2 (10.5–13.8)	7.5 (6.1–8.9)	9.5 (8.2–10.8)
Richest	15.9 (14.0–17.9)	11.0 (9.2–12.7)	11.8 (10.4–13.2)	15.9 (13.6–18.1)	14.1 (12.6–15.7)	12.8 (10.2–15.4)	8.0 (6.3–9.8)	16.5 (14.7–18.2)	11.0 (8.9–13.1)	15.6 (13.9–17.3)
**NSS 2004**										
Poorest	19.2 (16.7–21.8)	19.2 (16.3–22.0)	16.5 (13.6–19.5)	19.5 (16.9–22.0)	19.0 (16.4–21.7)	19.6 (16.8–22.3)	13.4 (11.4–15.4)	31.0 (27.1–35.0)	14.5 (12.2–16.7)	21.3 (18.5–24.2)
Poor	19.5 (17.0–22.0)	14.3 (12.3–16.3)	16.2 (13.7–18.8)	17.4 (15.2–19.6)	17.3 (15.1–19.4)	17.1 (14.7–19.6)	9.9 (8.4–11.5)	26.7 (23.7–29.7)	12.8 (10.8–14.9)	19.7 (17.3–22.0)
Middle	16.5 (14.7–18.3)	13.5 (11.8–15.2)	14.1 (12.0–16.1)	15.4 (13.7–17.0)	14.6 (13.0–16.3)	16.4 (14.4–18.3)	8.1 (7.0–9.3)	21.9 (19.6–24.2)	11.3 (9.7–12.9)	17.2 (15.3–19.1)
Rich	16.8 (15.0–18.6)	13.2 (11.6–14.8)	14.0 (12.3–15.7)	15.7 (14.0–17.5)	14.5 (12.9–16.0)	17.1 (15.1–19.1)	7.9 (6.8–9.0)	20.9 (18.9–22.9)	12.3 (10.6–14.0)	16.7 (15.0–18.4)
Richest	17.2 (15.6–18.9)	14.3 (12.7–15.9)	15.4 (13.9–17.0)	16.4 (14.5–18.3)	15.8 (14.3–17.3)	15.7 (13.9–17.5)	7.6 (6.5–8.6)	19.2 (17.4–20.9)	12.7 (11.0–14.4)	16.9 (15.4–18.5)
**NSS 2014**										
Poorest	23.4 (20.5–26.3)	19.6 (17.4–21.7)	20.5 (17.1–23.9)	21.4 (19.3–23.5)	17.8 (15.3–20.2)	24.7 (21.6–27.8)	12.3 (10.4–14.3)	39.8 (36.0–43.6)	11.7 (9.9–13.5)	24.3 (22.0–26.6)
Poor	14.4 (11.8–17.0)	12.3 (9.9–14.7)	13.3 (10.3–16.2)	13.3 (11.0–15.6)	12.4 (10.1–14.8)	14.7 (11.7–17.7)	6.7 (5.1–8.2)	23.5 (19.6–27.4)	7.9 (6.4–9.5)	15.3 (12.7–17.9)
Middle	18.3 (16.0–20.5)	14.2 (12.6–15.8)	16.7 (14.2–19.3)	15.9 (14.3–17.5)	14.8 (13.2–16.4)	19.2 (16.1–22.3)	7.4 (5.9–8.9)	24.1 (21.9–26.2)	9.8 (8.4–11.2)	18.7 (17.0–20.5)
Rich	20.3 (18.1–22.6)	12.5 (11.0–14.0)	16.8 (14.6–19.1)	16.2 (14.4–18.0)	14.8 (13.3–16.3)	21.3 (17.8–24.8)	6.8 (5.4–8.2)	24.5 (22.3–26.7)	9.0 (7.6–10.3)	18.9 (17.1–20.7)
Richest	21.8 (18.0–25.5)	16.4 (13.5–19.4)	19.2 (15.9–22.5)	18.5 (14.8–22.2)	18.6 (15.9–21.2)	20.4 (14.5–26.3)	6.4 (4.3–8.6)	23.8 (20.4–27.1)	10.5 (8.6–12.4)	20.7 (17.2–24.2)
Population under 60 years										
**NSS 1995–96**										
Poorest	6.5 (5.8–7.3)	6.1 (5.4–6.9)	4.0 (3.3–4.7)	6.9 (6.1–7.7)	5.7 (5.0–6.3)	7.7 (6.7–8.6)	5.3 (4.6–5.9)	8.8 (7.8–9.8)	5.8 (5.1–6.5)	8.0 (7.1–9.0)
Poor	5.8 (5.3–6.2)	4.9 (4.4–5.4)	3.6 (3.1–4.1)	5.7 (5.2–6.1)	5.0 (4.6–5.4)	6.0 (5.5–6.6)	4.3 (3.9–4.7)	7.5 (6.9–8.1)	4.6 (4.2–5.0)	7.5 (6.9–8.2)
Middle	7.5 (6.9–8.1)	6.5 (5.8–7.2)	4.8 (4.1–5.5)	7.8 (7.2–8.4)	6.8 (6.2–7.3)	7.7 (6.8–8.5)	5.3 (4.8–5.9)	9.0 (8.3–9.8)	6.1 (5.5–6.7)	9.6 (8.8–10.4)
Rich	7.8 (7.3–8.3)	7.0 (6.3–7.6)	5.5 (4.8–6.1)	8.4 (7.9–8.9)	7.1 (6.6–7.6)	8.1 (7.3–8.8)	5.6 (5.1–6.1)	9.1 (8.6–9.7)	6.3 (5.8–6.9)	10.2 (9.5–10.9)
Richest	11.9 (10.7–13.2)	10.7 (10.1–11.2)	9.2 (8.8–9.6)	13.9 (12.3–15.4)	11.0 (10.1–11.9)	12.3 (11.6–13.1)	8.3 (7.7–8.8)	13.1 (12.0–14.2)	9.2 (8.7–9.7)	15.3 (13.5–17.0)
**NSS 2004**										
Poorest	20.1 (18.8–21.4)	17.7 (16.4–18.9)	17.1 (15.5–18.7)	19.0 (17.8–20.2)	16.9 (15.8–18.1)	21.5 (20.1–22.9)	13.0 (12.0–13.9)	29.5 (27.7–31.2)	15.0 (13.9–16.1)	25.6 (24.0–27.2)
Poor	16.5 (15.5–17.5)	15.0 (14.1–15.9)	14.0 (12.8–15.3)	16.1 (15.2–16.9)	14.4 (13.6–15.3)	18.3 (17.2–19.3)	9.8 (9.1–10.4)	23.2 (22.0–24.4)	12.0 (11.3–12.8)	21.9 (20.7–23.1)
Middle	14.4 (13.5–15.2)	14.3 (13.4–15.2)	12.6 (11.4–13.7)	14.8 (13.9–15.7)	13.3 (12.5–14.1)	16.5 (15.5–17.4)	8.8 (8.2–9.4)	20.2 (19.1–21.4)	11.0 (10.3–11.7)	18.7 (17.6–19.8)
Rich	13.9 (12.5–15.4)	12.9 (11.9–13.9)	11.9 (10.3–13.4)	14.4 (13.3–15.4)	12.8 (11.5–14.0)	15.0 (13.9–16.1)	8.0 (7.3–8.7)	17.8 (16.2–19.4)	10.1 (9.0–11.3)	17.7 (16.4–19.0)
Richest	11.2 (10.6–11.9)	10.9 (10.3–11.6)	10.3 (9.7–10.9)	12.2 (11.4–13.1)	10.7 (10.1–11.2)	12.6 (11.8–13.3)	6.0 (5.5–6.4)	13.9 (13.2–14.5)	8.1 (7.6–8.6)	14.8 (14.0–15.6)
**NSS 2014**										
Poorest	21.0 (19.8–22.2)	17.3 (16.3–18.4)	19.5 (18.1–20.9)	18.9 (17.9–20.0)	18.3 (17.1–19.5)	19.4 (18.3–20.5)	11.2 (10.4–12.0)	35.4 (33.8–37.0)	13.3 (12.4–14.2)	25.3 (23.9–26.6)
Poor	15.5 (14.5–16.5)	13.2 (12.3–14.1)	14.5 (13.5–15.6)	14.2 (13.3–15.1)	14.5 (13.6–15.4)	14.0 (13.0–15.0)	7.4 (6.8–8.0)	25.2 (23.8–26.5)	10.1 (9.4–10.9)	19.2 (18.1–20.3)
Middle	14.6 (13.7–15.6)	12.7 (11.9–13.5)	13.1 (12.2–14.0)	13.8 (12.9–14.7)	13.6 (12.7–14.5)	13.7 (12.8–14.6)	6.5 (6.0–7.0)	21.7 (20.5–23.0)	9.4 (8.7–10.0)	17.7 (16.6–18.8)
Rich	13.2 (12.4–13.9)	11.0 (10.3–11.6)	11.4 (10.7–12.2)	12.4 (11.7–13.1)	12.0 (11.3–12.7)	12.1 (11.3–12.9)	5.3 (4.8–5.7)	17.9 (17.0–18.8)	8.3 (7.8–8.8)	16.1 (15.3–17.0)
Richest	14.3 (13.4–15.2)	11.7 (10.9–12.6)	12.6 (11.8–13.5)	13.4 (12.4–14.3)	12.8 (11.9–13.6)	13.4 (12.4–14.4)	5.7 (5.2–6.3)	15.7 (14.8–16.7)	8.8 (8.1–9.4)	16.6 (15.6–17.7)

OOPP: Out-of-pocket payments; MPCE: Monthly per capita consumption expenditure; CI: Confidence interval; CDs: Communicable diseases and nutritional disorders; Other diseases: Includes other diagnosed and undiagnosed ailments; NCDs: Non-communicable diseases and injuries.

Comparison with the younger population showed that the difference in OOPP between less and more developed states across quintiles was much higher for the older population in 2014. The younger population with similar capacity to pay were having a similar burden of OOPP both in the less and more developed states in 2014. Whereas, older population with similar capacity to pay had a higher burden of OOPP in less developed than the more developed states. The inequity in OOPP shares by gender was higher for the older and the younger population in 2014.

## 4 Discussion

This report provides evidence on the inequities in OOPP for hospitalisation in India over two decades up to 2014 and compares the older population with the population under 60 years. Six key findings relating to horizontal and vertical inequities in OOPP for hospitalisation and differentials emerge from this study. First, the older population had higher OOPP for hospitalisation and a greater increase in OOPP over two decades than the population under 60 years. Second, the increase in predicted mean OOPP for hospitalisation between 1995–96 and 2014 was higher for the poorest than the richest older population. Third, the increase in predicted mean OOPP for hospitalisation for the poorest and poor older population was higher between 1995–96 and 2004 than in the latter decade. Fourth, the vertical and horizontal inequity in OOPP for hospitalisation was higher for the older than the younger population in 2014. Fifth, the OOPP shares for hospitalisation were substantially higher in private hospitals, for non-communicable diseases and injuries, for those residing in less developed states and for males across MPCE quintiles in 2014, more so for the older population. Sixth, in 2014 the predicted absolute OOPP for hospitalisation was positively associated with economic status measured by MPCE quintiles but the predicted share of OOPP for hospitalisation in the household consumption expenditure was negatively associated with MPCE quintiles. This indicates that the OOPP for hospitalisation is a regressive means of financing healthcare in India.

Our study reveals some interesting findings based on the comparison of the older and the younger population. We found that the OOPP for hospitalisation was higher for the older than the younger population. Moreover, the older population had a greater increase in OOPP between 1995–96 and 2014. Higher expenditure on hospitalisation among older population is likely to stem from the fact that they have a higher burden of chronic diseases, more frequent hospitalisations and longer duration of stay in the hospital. The horizontal inequity in OOPP for hospitalisation by gender with the male having higher OOPP and consequently greater burden than female was more so for the older population than the younger population in 2014 which is in line with a recent study in India (Saikia and Bora, 2016). Lower socioeconomic status and the lack of financial empowerment among females are likely to be accentuated in older ages hindering the use of healthcare services resulting in lower expenditure than the male counterparts. Another interesting finding was that the disparity in OOPP by states improved for the younger population and deteriorated for the older population between 1995–96 and 2014. It can be inferred that the introduction of NRHM in 2005 with a major focus on the 18 less developed states had a positive impact on the health expenditures of the younger age groups in these states with no impact on those aged 60 years or more. Also, the rapid epidemiological and age transition in the less developed states might have added to the burden of morbidity among the older population in these states resulting in higher burden of OOPP for hospitalisation.

On the positive note, we found that the increase in OOPP for hospitalisation was lower in the latter decade for the poorest and poor MPCE quintiles both for the older and the younger population. This is an encouraging finding indicating that the comprehensive strategies, such as the Rashtriya Swastya Bima Yojana introduced in 2008, and a multitude of state-sponsored health insurance schemes in India have provided protection to the poor against high healthcare costs. Although these pro-poor programmes are far from achieving the goal of equity in healthcare financing, they seem to have a positive impact by protecting the poor households against catastrophic health expenditures (Mondal, 2015, Gupt et al, 2016). Providing insurance coverage is a means of protecting the households from large health expenditure without increasing public expenditure on health (Pal, 2010). The most recent move towards achieving universal health coverage prioritises financial protection and health security against impoverishment for the entire population of the country (Planning Commission of India, 2011).

The privatisation of healthcare services no doubt created enough provision for high quality and adequate services but they offered little relief to those who were constrained by resources in their ability to pay for these services, adding more to the dismal state of healthcare system in India (Bali and Ramesh, 2015). As expected, we found that the OOPP in private hospitals were substantially higher than the public hospitals. This is consistent with the finding from other studies in India (Bhat and Jain, 2006, Chandra et al., 2013). Additionally, the gap in the OOPP between private and public hospitals increased between 1995–96 and 2014, more so for the poor older population. The initiation of user fees in government facilities might have deterred the use of public hospitals and persuaded people to increase their utilisation of better quality private hospitals, ultimately increasing the cost of hospitalisation (Mondal, 2015). Prior to the health sector reforms in the 1990s, inpatient care was mostly available at public hospitals. Even though these services were fraught with quality issues, the poor could still access public inpatient care (Chaudhuri, 2012). The increasing dependence on private sector with a very weak regulation mechanism has led to a huge increase in healthcare costs in India (Balarajan et al., 2011). Strengthening of the public health facilities to provide quality care and regulating the private health facilities to limit cost escalation is the effective means of providing high-quality healthcare at the lowest possible cost.

Due to the higher burden of NCDs among the older population delivering healthcare is a big challenge for the healthcare system in India. We found that the cost of hospitalisation was higher for NCDs than CDs/other diseases and the rich were spending more in absolute terms on NCD hospitalisation at all time points. However, the burden of OOPP on hospitalisation for NCDs was higher for the poor than the non-poor older population in 2014. Another study in India showed that the wealthier spend more on the hospitalisation for cardiovascular diseases and diabetes than the poor (Rao et al., 2011). However, the NCD related OOPP for hospitalisation was catastrophic (out-of-pocket expenditure equalling or exceeding 10% of annual household consumption expenditure) for the poorest quintile (Tripathy et al., 2016).

The location of the hospitalised individual reflects the living conditions and has an impact on medical expenditures through health (O'Donnell et al., 2005). We found that the older population residing in the rural areas and in the less developed states had a higher burden of OOPP for hospitalisation. These findings are consistent with a previous study in India which found that rural areas and poor states experience a higher poverty headcount through OOPP mainly because a large proportion of their population is concentrated around the poverty line and hence even a small amount of OOP expenditure will push many households below the poverty line (Garg and Karan, 2009). Limited choice of local qualified providers, higher travel cost, including food and lodging for the escorts of the ailing household member and access issues causing delay in care seeking behaviour for conditions which then become more disabling and expensive to treat are some of the reasons for high burden of healthcare among rural households (Garg and Karan, 2009, Mondal et al, 2014). Higher rates of poverty, low per capita gross state domestic product, poor access to health infrastructure and professionals and low public health expenditure in less developed states results into households bearing the higher burden of OOPP for hospitalisation (Rao and Choudhury, 2012).

The regressive system of OOPP for hospitalisation in 2014 is an important finding from a policy perspective. A previous study in India showed that the OOPP for hospitalisation was regressive in 2004 (Mondal, 2013). OOPP in most countries is an especially regressive means of raising healthcare revenues indicating the inability and weakness of the healthcare system in financing and protecting its poor population from negative health shocks (Pannarunothai and Mills, 1997, Cisse et al., 2007, Akazili et al., 2011, Baji et al, 2012, Munge and Briggs, 2014, Bock et al, 2014, Rezapour et al, 2015). The regressiveness of OOPP also stems in part from the higher rates of sickness and medical consumption of the worse-off (Wagstaff and van Doorslaer, 1992). In Thailand, in spite of the provision of access to free care at public facilities for low-income households, the poor incurred higher costs of healthcare due to their preference for private facilities to avoid long delays involved in the referral system in public facilities (Pannarunothai and Mills, 1997). We found higher increase in the OOPP for the poor older population between 1995–96 and 2014. This might be the consequence of the introduction of the user fee in India during the eighth five-year plan (1992–97) under the umbrella of health sector reforms. Evidence suggests that the introduction of the user fee in public facilities increased the hospitalisation cost and resulted in large socio-economic inequalities in affordability of healthcare in India (Sen et al., 2002, Prinja et al, 2012, Mondal, 2015).

Given the low public spending on healthcare in India, the progressive nature of OOPP found in 1995–96 only reflects the capacity of the better-off to respond to healthcare needs by diverting resources from consumption while the poor forgo treatment to avoid the high cost of hospitalisation. A progressive nature of healthcare cannot be a positive indicator of fairness in financing if the poor population use less care despite a greater burden of illness (Chaudhuri and Roy, 2008). A study in Srilanka found that the burden of out-of-pocket health payments did not vary substantially with the ability to pay reflecting that the poor face more hardships and financial impoverishment due to high healthcare costs (Kumara and Samaratunge, 2016). On the contrary, a heavily subsidised public sector and a user charged private sector produced a progressive health financing system in Malaysia (Yu et al., 2008). The distribution of OOPP also depends largely on the level of development of a country. In high-income economies with widespread insurance coverage, OOPP absorbs a larger fraction of the resources of low-income households whereas, in poor economies, it is the better-off that spend relatively more out-of-pocket (O'Donnell et al., 2008).

One of the limitations of this study is that the level of disaggregation in collecting data on household monthly consumption expenditure across surveys was not similar. The level of disaggregation in NSS 2004 and NSS 2014 was less than in NSS 1995–96. A lower level of disaggregation can potentially lead to a lower estimate of total consumption expenditure. However, in this case, the difference between the mean household consumption expenditure in the health surveys was similar to the mean expenditure in the household consumer surveys that collected more detailed consumption expenditure. Second, the hospitalisation expenditure was collected in a reference period of 365 days which might have introduced some recall bias in reporting. Third, we concentrated only on direct medical and non-medical expenditures, not taking into account the indirect burden due to hospitalisation episodes like work loss, worker replacement and reduced productivity from illness and disease which might have underestimated the burden of healthcare cost.

In spite of the limitations, this study provides a comprehensive overview of the horizontal and vertical inequities in OOPP for hospitalisation of the older population comparing it with younger age groups in India over two decades. Moreover, the use of regression methods provides a more accurate description of the nature of inequities prevailing in the distribution of OOPP rather than the summary measure of progressivity or horizontal inequity (Cisse et al., 2007). The merit of this study lies in the use of more comprehensive data on health expenditure available from health surveys to calculate the OOPP and its burden at the individual level. Additionally, since we restricted our analyses only to hospitalised individuals we can infer with greater confidence that the observed difference in OOPP by economic status was due to the inadequacy of the healthcare system rather than the differences in underlying health status (Roy and Howard, 2007).

In conclusion, we can say that the older population in India has higher OOPP for hospitalisation and also greater inequity than the younger population. Given the rising cost of hospitalisation and the corresponding higher burden on the poor older population, health policy in India should prioritise universal health coverage, promote risk pooling mechanisms and most importantly increase the public expenditure on health. These measures will be instrumental in reducing the burden of OOPP for hospitalisation among older population in India.

## Authors’ contributions

AP extracted the data, conducted statistical analyses, interpreted the findings and wrote the first draft of the manuscript. GBP and LC contributed to the initial concept of the paper. GBP, LD and LC guided the interpretation of findings and provided critical comments on the manuscript for intellectual content. GBP and LD guided the statistical analyses. AP revised the manuscript under the guidance of LD in the review process and all authors approved the final version of the manuscript.

## Figures and Tables

**Appendix Table 1:** Predicted mean annual out-of-pocket payments for hospitalisation in India, NSS 1995–96.

MPCE quintile	Predicted mean annual OOPP for hospitalisation (US$) (95% CI)			
	Older population		Population under 60 years	
	NSS 1996^a^	NSS 1995–96^b^	NSS 1996^a^	NSS 1995–96^b^
Poorest	44 (22–67)	33 (24–41)	32 (24–39)	34 (30–38)
Poor	36 (29–43)	38 (33–44)	39 (35–43)	43 (37–49)
Middle	52 (41–63)	81 (66–96)	66 (58–73)	68 (63–73)
Rich	103 (85–121)	107 (94–120)	89 (80–97)	87 (82–92)
Richest	275 (241–309)	296 (262–329)	220 (190–250)	214 (195–233)
Total	117 (107–127)	121 (113–129)	103 (95–110)	97 (93–102)

CI: Confidence interval; NSS: National Sample Survey; MPCE: Monthly per capita consumption expenditure; OOPP: Out-of-pocket payment; US$: United States dollars.

a January–June 1996.

b July 1995–June 1996.

**Appendix Table 2:** Ratio of mean annual out-of-pocket payments for hospitalisation in NSS 2014 to NSS 1995–96, adjusted for inflation using GDP deflator and CPI in India.

MPCE quintile	Ratio of mean annual OOPP for hospitalisation in NSS 2014 to NSS 1995–96			
	Older population		Population under 60 years	
	GDP deflator adjusted prices	CPI adjusted prices	GDP deflator adjusted prices	CPI adjusted prices
Poorest	4.6	3.7	5.0	4.0
Poor	4.5	3.6	4.3	3.4
Middle	2.5	2.0	3.2	2.5
Rich	3.2	2.5	2.7	2.2
Richest	2.4	1.9	1.6	1.3
Total	2.4	1.9	1.9	1.5

OOPP: Out-of-pocket payments; GDP: Gross domestic product; CPI: Consumer price index; MPCE: Monthly per capita consumption expenditure; NSS: National Sample Survey.

**Appendix Table 3:** Selected socio-economic characteristics by monthly per capita consumption expenditure quintiles for the hospitalised population under 60 years in India, NSS 1995–96, NSS 2004 and NSS 2014.

	Poorest	Poor	Middle	Rich	Richest	All
**NSS 1995**–**96**						
Female (%)	50.9	53.0	47.5	47.8	47.4	48.5
Illiterate (%)	63.2	54.7	47.5	42.1	27.3	41.1
Rural (%)	83.0	84.1	78.1	70.3	49.8	67.2
Less developed states^a^ (%)	36.0	30.9	32.2	32.0	25.7	29.9
Hospitalised for NCDs^b^ (%)	26.1	27.4	31.3	32.3	39.9	33.73
Used private hospital (%)	33.1	36.4	51.5	55.4	66.4	54.36
Mean annual OOPP (US$) per hospitalised person (SD)	39 (54)	47 (168)	77 (111)	99 (140)	302 (692)	156 (442)
Mean annual household consumption expenditure (US$) per capita (SD)	185 (26)	246 (16)	306 (19)	389 (31)	697 (373)	450 (296)
Hospitalised (%)	0.52	0.83	1.06	1.51	2.34	1.25
**NSS 2004**						
Female (%)	49.4	48.5	49.4	48.6	48.0	48.7
Illiterate (%)	56.3	49.3	42.8	35.6	22.7	38.6
Rural (%)	91.9	86.3	79.7	65.3	41.8	69.0
Less developed states^a^ (%)	45.2	38.0	34.8	31.3	23.3	32.9
Hospitalised for NCDs^b^ (%)	41.2	43.9	48.4	49.4	50.9	47.6
Used private hospital (%)	42.6	53.3	56.6	63.5	72.4	60.0
Mean annual OOPP (US$) per hospitalised person (SD)	118 (202)	142 (249)	169 (318)	225 (529)	306 (776)	207 (516)
Mean annual household consumption expenditure (US$) per capita (SD)	167 (31)	236 (16)	300 (21)	395 (37)	749 (452)	413 (317)
Hospitalised (%)	1.5	1.7	2.0	2.4	3.0	2.1
**NSS 2014**						
Female (%)	52.9	52.5	49.3	50.4	52.3	51.4
Illiterate (%)	46.1	40.6	37.6	32.8	21.2	34.1
Rural (%)	88.9	80.8	74.7	63.3	39.1	66.3
Less developed states^a^ (%)	63.9	51.0	38.1	32.1	21.4	38.5
Hospitalised for NCDs^b^ (%)	53.6	52.0	57.5	54.7	59.1	55.7
Used private hospital (%)	42.1	51.1	59.2	65.9	79.5	62.0
Mean annual OOPP (US$) per hospitalised person (SD)	194 (383)	200 (433)	244 (620)	272 (522)	478 (1,056)	294 (695)
Mean annual household consumption expenditure (US$) per capita (SD)	225 (43)	328 (27)	423 (29)	558 (55)	1042 (486)	564 (384)
Hospitalised (%)	1.81	2.15	2.6	3.03	3.83	2.6

a Includes eight empowered action group states (Bihar, Jharkhand, Madhya Pradesh, Chhattisgarh, Uttar Pradesh, Uttaranchal, Odisha and Rajasthan), 8 north-eastern states (Assam, Arunachal Pradesh, Manipur, Mizoram, Meghalaya, Nagaland, Sikkim, Tripura), Himachal Pradesh and Jammu and Kashmir.

b Based on Global Burden of Disease (2013) classificationNSS: National Sample Survey; OOPP: Out-of-pocket payments; NCDs: Non-communicable diseases and injuries; SD: Standard deviation.
